# A Rare Case of Mastoid Process Osteoma Presenting During Puberty: A Case Report

**DOI:** 10.3390/reports8020081

**Published:** 2025-05-26

**Authors:** Aleksandrina Topalova-Shishmanova, Georgi Pavlov

**Affiliations:** 1 Department of Ear, Nose and Throat Diseases, Faculty of Medicine, Medical University of Plovdiv, 4000 Plovdiv, Bulgaria; 2 Department of Anesthesiology, Emergency and Intensive Care Medicine, Medical Faculty, Medical University of Plovdiv, 4000 Plovdiv, Bulgaria; georgi.pavlov@mu-plovdiv.bg

**Keywords:** osteoma, mastoid osteoma, temporal bone

## Abstract

**Background and Clinical Significance**: Osteomas of the mastoid process are extremely rare tumors. In their development, they are usually asymptomatic, they can manifest with cosmetic deformity, pain, hearing loss, and weakness of the facial nerve. **Case Presentation**: We present a clinical case of a 13-year-old girl with complaints of swelling in the area behind the left pinna, which was painless but created a cosmetic defect and an unpleasant sensation. She reported no pain in the ear and no hearing loss. An otorhinolaryngological examination, an audiometry of the patient, and a computed tomography of the head, temporal bones, and middle and inner ear were performed. The test results showed that hearing was not affected, and the tumor in the area of the mastoid process was approximately 3 cm in diameter, sitting “on top” of the mastoid process. Extirpation of the osteoma was performed with a retroauricular approach. The patient had a short postoperative period without any complications. **Conclusions**: Mastoid osteomas are rare, benign, slow-growing, and frequently asymptomatic bone tumors. Other bone lesions of the mastoid region should be ruled out in the differential diagnosis. Surgery is the treatment of choice and should be performed in the presence of symptoms or for cosmetic reasons.

## 1. Introduction

Osteomas are benign and most often asymptomatic tumоrs. They can be classified according to their localization, size, and degree of extension. In the head and neck region, they occur most frequently in the ethmoid and frontal bones. Osteomas of the temporal bone can affect the external auditory canal, squamous part, mastoid process, internal auditory canal, middle ear, Eustachian tube, petrous apex, and styloid process. It occurs more frequently in female patients, primarily during the second and third decades of life, and is uncommon during puberty [1].

Mastoid osteomas are extremely rare tumors accounting for about 0.1–1% of all osteomas in the head and neck region [2]. By 2019, only 200 clinical cases of mastoid osteoma had been published [3].

They represent well-differentiated bone tissue with a laminar and well-defined surface originating most often from the cortex of the mastoid process and projecting outwards to the skin. Osteomas can be histologically classified as compact, spongy, or mixed.

In the case of the presence of osteomas of the craniofacial skeleton, including the mastoid process, it is essential to suspect them in the differential diagnosis of “Gardner” syndrome. Gardner syndrome is associated with colorectal and extracolonic pathology, and the recognition is crucial for appropriate clinical management [4].

The clinical course is most often asymptomatic with no pain, but as they grow, they could cause cosmetic deformities associated with a change in the position of the auricle, as well as swelling and discomfort. Occasionally, they may cause hearing loss.

**Figure 1:**
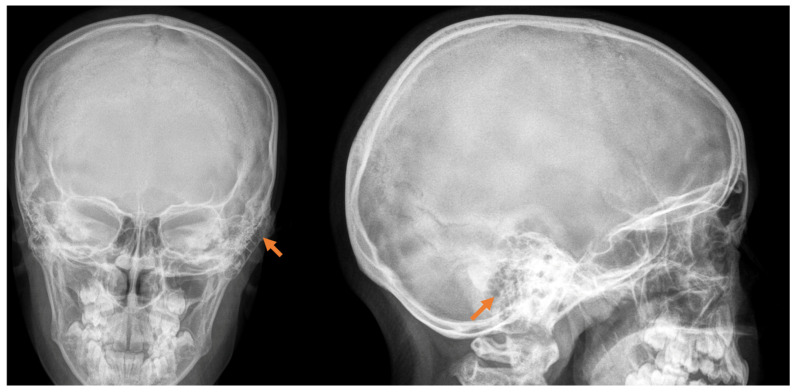
Radiological examination in front and side view, with the detection of a slight deformation along with bone growth in the cortical area of the mastoid process, pointed with orange arrow.

The diagnosis is made by performing a computed tomography scan, with a clear presentation of the size, density, and boundaries of spread (Figure 1). In the presented clinical case, we establish a mastoid process osteoma with slow progression for a period of 6 years, creating only a cosmetic defect in the retroauricular area.

## 2. Case Presentation

This case study involves a 13-year-old female patient who, at the age of 7, noticed a slight painless swelling in the left retroauricular region. The first diagnosis of the tumor was made with radiography.

**Figure 2:**
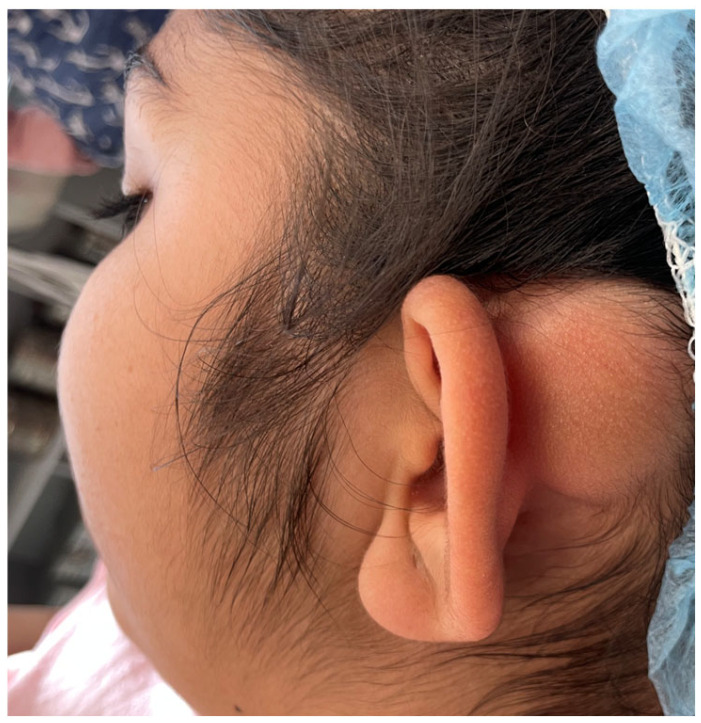
Visualization of the position of the osteoma in the left occipital region with a pronounced skin deformity of the auricle protruding forward, like a “floppy” ear.

Over a six-year period, it had significantly increased in size with visible cosmetic deformity. The patient had mild pain, especially when lying on the same side, without any other functional disturbances (Figure 2).

We did not identify any bone deformities or clinical signs of desmoid tumors. The patient has no family history of Gardner syndrome or hereditary multiple exostoses.

**Figure 3:**
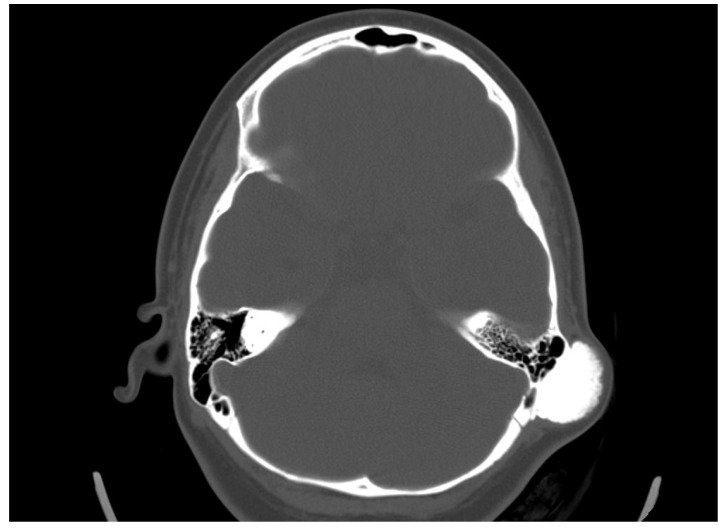
Axial CT scan revealing an osteoma in the cortex of the mastoid process, with no involvement of the surrounding cellular structures.

She was admitted to the ENT department, and an otorhinolaryngological examination was performed. In the retroauricular area, tumor formation was palpated, and the overlying skin was mobile with no retraction. Otoscopy did not detect any abnormalities in the external auditory canal and the tympanic membrane. The audiological examination did not indicate any hearing loss. A computed tomography scan was conducted. In the axial plane, a bone tumor formation with a diameter of 19/31 mm in the mastoid process was detected. There was a passive dislocation of the soft tissues with no invasion (Figure 3).

Following thorough discussions with the patient and her family, a decision was reached to proceed with surgical treatment, taking into account the patient’s age, gender, and the significant cosmetic deformity.

**Figure 4:**
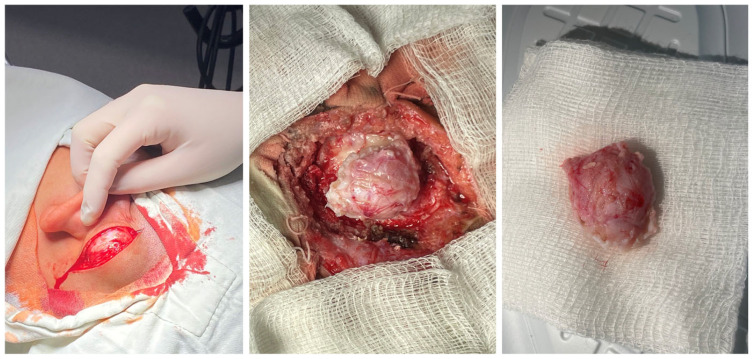
External retroauricular access with a dissection of the bone tumor using a high-speed drill and complete (en bloc) removal.

Using a retroauricular approach under general endotracheal anesthesia, a skin incision was made, and the tumor was immediately accessed. The bone tumor was meticulously dissected and excised with the aid of a high-speed drill. A few individual mastoid air cells were observed in minimal volume near the cortex (Figure 4).

Histological examination confirmed the diagnosis of compact osteoma. The postoperative course was uneventful. No local recurrence was observed at the last follow-up visit, which occurred one year after the operation.

## 3. Discussion

Temporal bone osteomas are extremely rare benign mesenchymal tumors with slow growth and often have an asymptomatic clinical course in the early stages [5]. They most often affect females in the second and third decades of their lives. The clinical manifestation during puberty is extremely rare, which makes our clinical case even more uncommon.

The etiology of osteomas remains unclear. Congenital predisposition, inflammation, and trauma are considered possible causes, but as for the mastoid process, it usually remains idiopathic [6]. In the presented clinical case, it was probably a congenital osteoma since the patient had not experienced any previous infections or trauma in this area.

Craniofacial osteomas are also associated with Gardner’s syndrome. It is characterized by colorectal polyposis carrying a high potential for malignancy, including the presence of multiple facial skeleton osteomas, subcutaneous fibromas, desmoid tumors, and epidermal cysts. A detailed medical history is of utmost importance for the association of osteomas with other clinical syndromes [7].

The clinical course of osteomas is often asymptomatic, and they are usually diagnosed incidentally. Depending on the localization, they may present with facial deformities, ocular manifestations (exophthalmos), and headaches [7]. The diagnosis of osteomas can be made by skull radiography/skull X-rays (occipitomental projection, also known as Water’s view) or computed tomography without contrast enhancement, which has many more advantages. Computed tomography with 3D reconstructions allows for the detailed visualization of osteomas and their specific localization in relation to other vital structures (the orbit, anterior, medial, and lateral skull base, facial nerve and intracranial structures). It can also determine the attachment of the osteoma to the cortex in the case of mastoid processes—pedunculated or sessile [8]. The study is also useful in the differential diagnosis of osteoma and other mastoid bone tumors, especially osteosarcoma, bone metastases, multiple myeloma, giant cell tumor, Paget’s disease-associated lesions, or fibrous dysplasia [1,6,7,9]. Signs suggesting a malignant lesion are rapid growth, local pain, and a poorly differentiated, heterogeneous, osteolytic appearance on CT [9]. Mastoid osteomas are slow-growing and can seldom be indicated for surgical treatment in the early stages. The cosmetic defect that they would create is the most common reason for surgical removal. Osteoma removal is performed through a retroauricular incision exposing the tumor and its radical removal using a curette or burr. The presentation of intact mastoid cells is normal and does not pose a risk. Sometimes, a slightly prominent sinkhole can possibly occur postoperatively in this area. Histological examination is important to confirm the diagnosis and specify the histological type—compact, spongy, or mixed. Compact or combined is most often found, and spongy is much less common [10].

## 4. Conclusions

Mastoid process osteoma is an extremely rare, slow-growing benign tumor. It is most often asymptomatic but can cause a cosmetic defect in the retroauricular area. Osteomas of the head bones can also be associated with conditions such as Gardner’s and Paget’s syndromes. Computed tomography of the temporal bone is the diagnostic method of choice for these neoplasms, providing detailed information about the size, density, and involvement of surrounding structures. Malignant transformation of osteomas is uncharacteristic. Surgical treatment is indicated for symptomatic patients and those with cosmetic defects.

This case report contributes to the small amount of documented mastoid osteomas, especially in younger patients. It is important to note that the resulting deformity and psychological impact, especially in an adolescent, highlight the importance of surgical intervention not only for medical but also for psychosocial reasons. The successful outcome following complete excision and the absence of recurrence over a one-year follow-up period further support surgical management as a definitive and curative option.

Moreover, the lack of family history or other associated lesions in this patient ruled out syndromic associations, but this case emphasizes the necessity of thorough clinical evaluation and appropriate screening to exclude conditions like Gardner syndrome when craniofacial osteomas are detected. The first choice of radiology method remains the high-resolution CT, which is essential to understand where the lesion starts, how far it spreads, and how it relates to nearby important anatomical structures.

In conclusion, despite their rarity, mastoid osteomas should still be considered in the differential diagnosis of retroauricular masses. Early detection, precise imaging, and careful surgical planning are essential elements for optimal patient care. Regular reporting of such rare cases is vital for expanding clinical awareness and improving diagnostic and therapeutic strategies in ENT practice.

## Data Availability

The original contributions presented in this study are included in the article. Further inquiries can be directed to the corresponding author.
